# Analysis of Security Issues in Wireless Body Area Networks in Heterogeneous Networks

**DOI:** 10.3390/s22197588

**Published:** 2022-10-06

**Authors:** Somasundaram Muthuvel, Sivakumar Rajagopal, Shamala K. Subramaniam

**Affiliations:** 1 Department of Electronics and Communication Engineering (ECE), R.M.K. Engineering College, Kavaraipettai 601206, Tamilnadu, India; 2 Department of Sensor and Biomedical Technology, School of Electronics Engineering (SENSE), Vellore Institute of Technology, Vellore 632014, Tamilnadu, India; 3 Department of Communication Technology and Networks, Universiti Putra Malaysia, Serdang 43400, Malaysia

**Keywords:** Body Area Network (BAN), eHealthcare, heterogeneous networking, network security, game theory

## Abstract

Body Area Network (BAN) is one of the most important techniques for observing patient health in real time and identifying and analyzing diseases. For effective implementation of this technology in practice and to benefit from it, there are some key issues which are to be addressed, and among those issues, security is highly critical. WBAN will have to operate in a cooperative networking model of multiple networks such as those of homogeneous networks, for the purpose of performance and reliability, or those of heterogeneous networks, for the purpose of data transfer and processing from application point of view, with the other networks such as the networks of hospitals, clinics, medical experts, etc. and the patient himself/herself, who may be moving from one network to another. This paper brings out the issues related to security in WBAN in separate networks as well as in multiple networks. For WBAN working in a separate network, the IEEE 802.15.6 standard is considered. For WBANs working in multiple networks, especially heterogeneous networks, the security issues are considered. Considering the advancements of artificial intelligence (AI), the paper describes how AI is addressing some challenges faced by WBAN. The paper describes possible approaches which can be taken to address these issues by modeling a security mechanism using various artificial intelligence techniques. The paper proposes game theory with Stackelberg security equilibrium (GTSSE) for modeling security in heterogeneous networks in WBAN and describes the experiments conducted by the authors and the results proving the suitability of the modeling using GTSSE.

## 1. Introduction

The Body Area Network placed an important role in healthcare application to monitoring patient health in real time. The sensor devices are used to observe the patient health and identifying various disease. For effective implementation of this technology in practice and to benefit from it, there are some key issues which are to be addressed. Among them, security issue is one of the them, where research is being conducted. WBAN uses wireless sensors specifically for use within or on the human body and captures many parameters, so it enables various applications related to health or medical or even general purposes. 

IEEE has published the IEEE 802.15.6 standard for sensor networks, which has been developed by the Task Group IEEE 802.15.6 [1]. The IEEE 802.15.6 standard [2] has been specified for making short-range communication which is approved by the authorities for utilization in that is utilized in industry and medicine. The sensor network has specific characteristics such as low power, quality of services and a 10Mbps transfer rate. This process ensures the low specific absorption rate (SAR). The standard is available as a document in [2]. 

**Figure 1:**
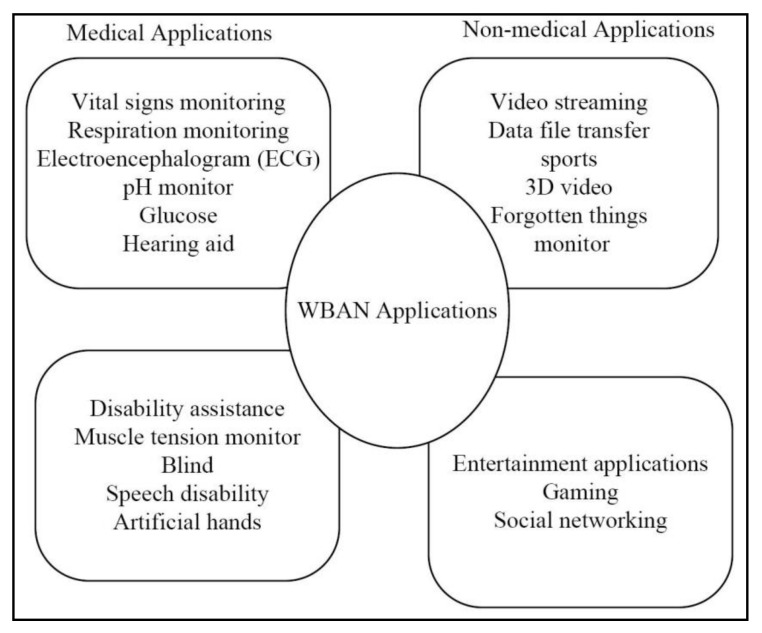
IEEE 802.15.6 standard-based body sensor networks applications.

**Figure 2:**
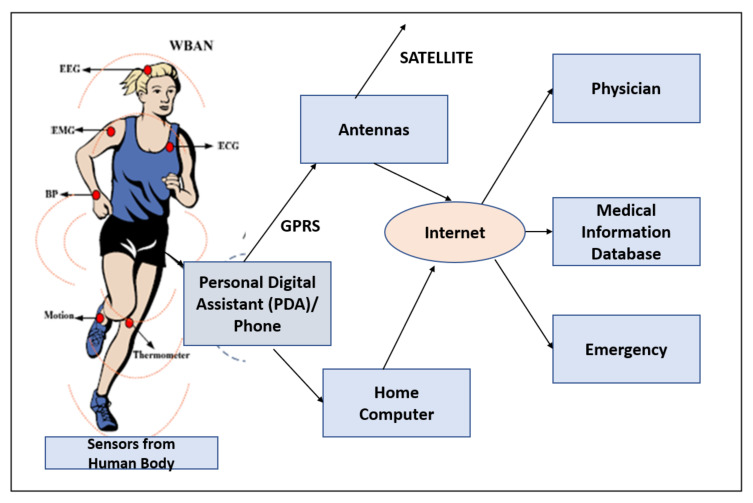
Structure of Body Area Networks.

The WBAN applications addressed by the IEEE 802.15.6 standard are either non-medical and medical applications as given in Figure 1. Figure 2 [3] gives a pictorial description of the WBAN structural design for various applications. 

This paper brings out the issues related to security in WBAN in separate networks as well as in heterogeneous networks and possible approaches which can be taken to address them. Section 2 defines the security issues in a WBAN in a single network, and Section 3 defines the security issues in WBAN in multiple networks. Section 4 describes the various artificial techniques which can be used to effectively model the security issues in WBAN, and Section 5 gives a conclusion of the analysis of different techniques and proposes one of them for effective modeling with higher security.

## 2. Security Problems in a WBAN in a Single Network

In view of the WBAN applications, various research issues [4] have to be taken into account for effective and reliable usage of WBAN in the intended applications. They are design of radio frequency (RF) wireless systems, channel modeling, quality, antenna design, and reliability, PHY protocol design, MAC protocol design, dissimilar networks connectivity, security, monitoring and privacy. Among the several issues, security issues [5] are more considerable because it completely reduces the sensor-based communication. The main security requirements are listed as follows [3].

- Data truthfulness, confidentiality, freshness, authentication, security administration and availability.

Many new technology solutions are emerging and they have both advantages and disadvantages. The detailed security requirements are explained in [3]. Attacks on WBAN can be at various layers of data communications, and the defenses against them are classified in the Table 1 below.

### 2.1. IEEE 802.15.4-Based Body Sensor Networks Security Framework

**Figure 3:**
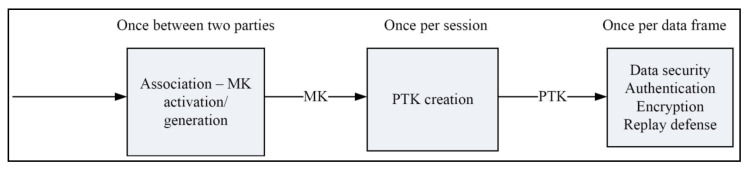
IEEE 802.15.6 standard security structure.

Security requirements are attained by using the IEEE 802.15.4 standard in body sensor networks. This framework process the low-data applications because of the minimum power standard. It is meant for lower network layers of a type of wireless personal area network (WPAN) that is utilized to making the effective communication between the devices with high speed and low cost. This is different from more end-user-oriented approaches of personal area networks, for example, Wi-Fi [3]. The IEEE standard is very simple and effective for communication and is similar to the body sensor networks because it requires a lower power cost and data rate, etc. Table 2 and Table 3 and Figure 3 (“Security in IEEE 802.15.4”) show how the security has been implemented in the standard [6].

Table 3 (“Security in IEEE 802.15.6 standards”) shows the three levels of security. [1] The security structure is as per IEEE standard 802.15.4 with necessary changes.

### 2.2. Security Problems and Solutions

**Figure 4:**
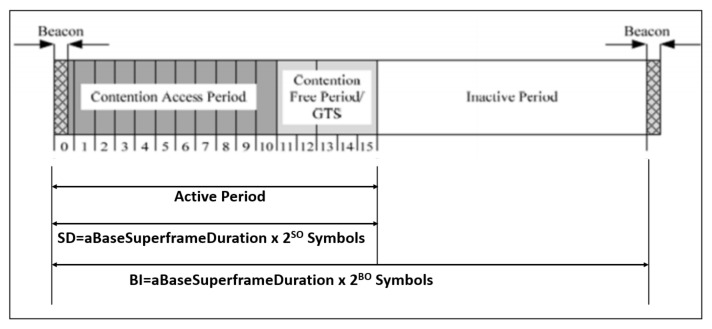
Beacon enable mode-based IEEE 802.15.4 communication structure.

The protocol well defined in the IEEE 802.15.4 MAC has some security issues to be addressed. The protocol consists of a super frame configuration containing active and inactive periods, as illustrated in Figure 4. [7] The system comprises three constituents such as beacon, contention access period (CAP) and contention-free period (CFP). The controller communicate with the nodes at the time of rest period (active and inactive). This communication establishes only seven GTS slots to minimize the traffics. During the beacon communication mode, CA/CSMA protocol is utilized to make the effective communication. Finally, the unslotted CSMA/CA protocol is utilized in the non-beacon mode communication.

The IEEE 802.15.4 framework is influenced by several attacks; therefore, different GTS slots are required to enable the secure communication when compared to the weak and random attacks. Hence, the framework is created to manage the various traffics and vulnerable attacks [7] during the communication. To attain the issues, a different security structure is created to predict the intermediate and backoff attacks. The introduced methods should manage the security by increasing the network throughput. In addition to this, different security approaches are developed for deciding the sender backoff windows. The backoff windows helps to identify the attacks and penalize the adversaries effectively on the receiver side. To consider these factors, a game-theoretic approach is utilized to identify the threats and attacks effectively [3,8].

## 3. Security Issues in WBAN in Multiple Networks

The wireless communication for Body Area Networks has been increasing in terms of traffic and application recently. This has led to the use of WBAN networks in collaboration with multiple networks broadly called Cooperative Networks. They are of two types:

- Multiple similar or homogeneous WBAN networks collaborating and cooperating from performance and reliability points of view: the objective is to communicate the data more effectively from source to destination.
- WBAN network communicating with other types of networks of different types from the application of view: The goal is to transfer the data to other networks of other types by transferring the data for further processing. As a part of this, there will be a scenario by which the WBAN consisting of the patient, sensors and data gathering may be mobile and will communicate through different and heterogeneous networks during the data gathering.

### 3.1. Cooperative Networks for Performance and Reliability

Jie Dong and David Smith, in their paper, “Cooperative Body-Area-Communications: Enhancing Coexistence Without Coordination Between Networks”, analyzed the co-occurrence of the various mobile body sensor networks. The sensor networks uses the cooperative communications to manage the effective communication [9].

In general, BAN should be responsible for extremely reliable communication with little transmission power. As there may be large path losses for single link star topology [10,11], IEEE 802.15.6 provides two-hop cooperative communications as an option [12], which has been found to give significant performance benefits using either narrow-band [13,14,15,16] or ultra-wideband layers [17]. However, as the WBAN is being used extensively with multiple WBANs being closer to each other, the coexistence is becoming an issue. IEEE 802.15.6 standard requires that a system should maintain reliable performance with up to 10 WBANs co-located in a 6 × 6 × 6 m space. So, a new technology called Cooperative Network Coding (CNC) addresses this issue by providing effective decode-forward protocol with two relays, two-hop links and selection conjoining at hub (or gateway device) using time-division multiple-access (TDMA) which supports both inter- and intra-network interactions [18]. It is found that this approach provides significant performance improvement and increased the throughput and network reliability [9,19].

### 3.2. Cooperative Networks for Data Transfer and Processing

**Figure 5:**
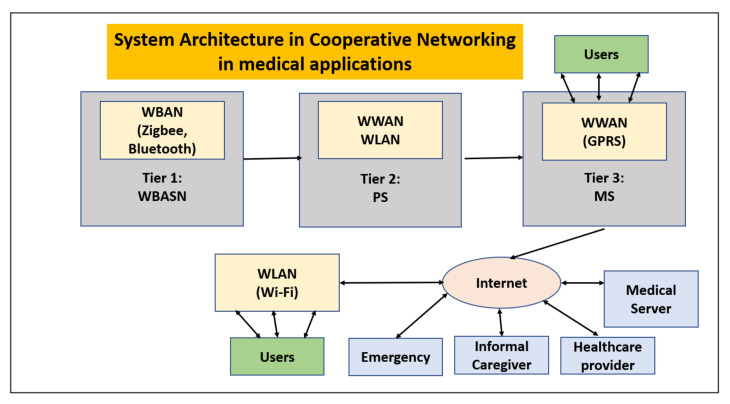
System architecture in cooperative networking in medical applications.

Xigang Huang has conducted extensive research on the Efficient Cooperative Communications for Wireless Body Area Networks where the networks are of different types and, hence, called heterogeneous networks [18]. Cooperative networking is highly relevant in medical applications as the WBAN devices have to transmit data across various networks, as given in Figure 5. Additionally, if the WBAN devices are attached to the patient who himself/herself is mobile (for example, moving in a vehicle or ambulance to the network of the hospital, etc.), then the WBAN communications should be communicated across different networks. The body sensors are classified as medical and non-medical sensors, and as biosensor and motion sensors. Furthermore, the traffic is classified into video stream [20], parameter stream and wave-form real-time stream.

The sensors are utilized in healthcare applications for the following reasons: high-priority data transmission; time- and veridical-based data acquisition; channel characterization and patient mobility; time-varying and dynamic environment. In addition to this, the sensors are utilized to record ECG, and EEG-related uncompressed videos also record human postures and limb movement.

In a cooperative network, as above, where data has to be transferred and processed across multiple networks, and the networks have to be cooperative, issues arise in proper design of the protocols for the routing. Such routing typically is taken care of at the MAC layer level. So, the issues in this level are:

- Designing network MAC protocols to enable WBANs to adaptively and intelligently balance the QoS requirements and unique constraints;
- Designing cooperative communication protocols for WBANs;
- Combining the minimum energy route and low duty cycle scheduling to maximum network lifetime of WBANs while satisfying QoS requirements, which is a challenging issue.

**Figure 6:**
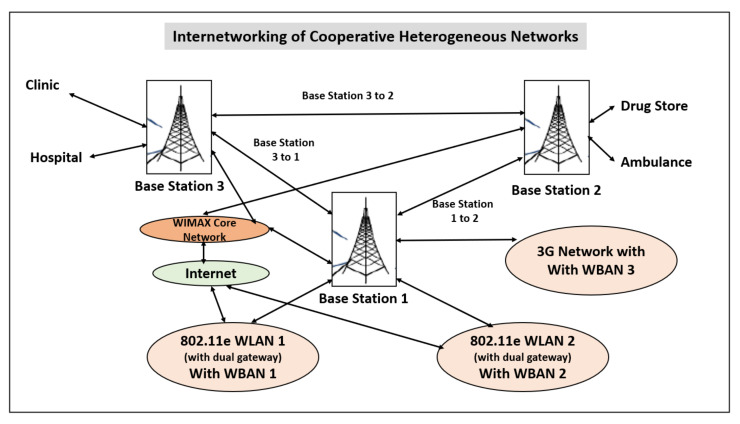
Internetworking of cooperative heterogeneous networks.

Internetworking of Cooperative Heterogeneous Networks: Issues [18] [Figure 6].

The research shows that there are many challenges to be overcome in the type of cooperative networks which are heterogeneous:

- It is obvious that allocating bandwidth efficiently when integrating heterogeneous wireless networks is a challenge.
- How to provide medical QoS consistently over integrated Wi-Fi/WiMAX wireless networks is a challenge in the research.
- How to efficiently manage radio resources, manage scheduling, and control connection admission are still open issues in WiMAX networks; they are also critical in integrated Wi-Fi/WiMAX wireless networks for E-health services.
- Hand-over management for seamless integration of wireless networks and for providing continuous E-health services for mobile users may be one of the most challenging issues, due to the transfer of vital medical information through dynamic wireless channels and networks.
- A challenging issue is how to efficiently manage the spectrum to accommodate different applications using cognitive radio technology.
- How to design a source-aware secure mechanism within WBAN and with heterogeneous networking is also a challenge.

Some of the mechanisms being followed in cooperation are:

- With the dimensions DF and AF:
- Fixed relaying;
- Selection relaying;
- Incremental relaying;
- Incremental transmission relay selection (ITRS);
- Multi-hop with relay selection (MHRS).

There is no cooperative protocol which has been designed for WBANs. In cooperative networking, WBANs or sensor networks must be integrated into other heterogeneous networks such as cellular/Wi-Fi networks in ubiquitous computing.

### 3.3. Need for Heterogeneous Networks (HetNet) for WBAN

As described above, WBAN requires to operate in a heterogeneous network to effectively transfer data and for further processing. With this need, there are many issues which are to be examined for effective implementation of heterogeneous network. This has opened up many research issues [21].

### 3.4. Cognitive and Cooperative Communications for HetNet

X. Zhang and Y. Gaoy et al., in their paper, “Cognitive and Cooperative Communications in Wireless Heterogeneous Networks (HetNet): Current Status and Technical Perspectives”, have conducted extensive research on HetNet and have proposed a structural design for the integration of cognitive networks and cooperative communications in wireless HetNet [22]. Based on the proposed architecture several techniques related to the integration of cognition and cooperation are evaluated. Techniques such as cognitive relay network, geolocation-based cognition and cognitive and cooperative gateway can be effectively applied. Simulations and analysis have been conducted showing that the combination of cognition and cooperation can significantly improve the system performance.

### 3.5. Mobile Cloud Computing (MCC) Enabled WBAN Architecture

**Figure 7:**
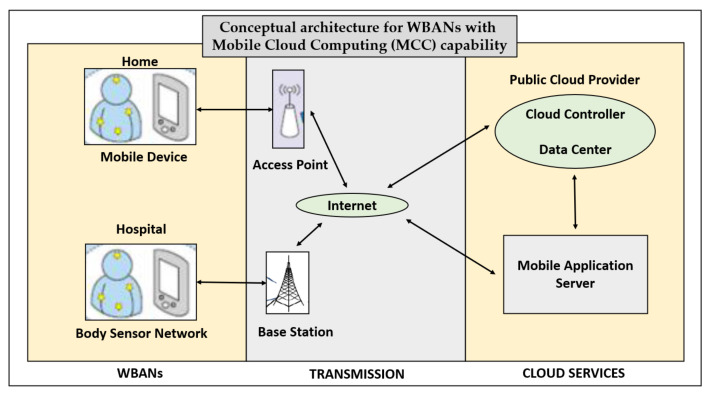
Conceptual architecture for WBANs with MCC capability.

With the advent of Cloud Computing and Mobile Computing, the WBAN data gets created, transmitted to, processed at, and stored at various nodes in a network, which includes Cloud Computing environment and Mobile Computing front-end interfaces. So, this raises a scenario of another type of heterogeneous networks of WBANs. Figure 7 depicts such a MCC-capable framework for a pervasive healthcare system, and it is studied in [23].

### 3.6. Protocols for Cooperative Communications: CoopMAC

Cooperation means that a virtual antenna array is formed when numerous nodes in a sensor network collaborate to form a computer-generated antenna array. Across the nodes of the network deprived of separate nodes necessarily having numerous antennas. It is basically different from multi-hop communications in which the destination only receives one version of the message from the source.

Some basic aspects requiring study and research include: theoretical tools to aid in the design of cooperative networking systems, as well as effective incentive mechanisms for the nodes to cooperate, and new protocol designs at the physical and network (especially MAC) level for cooperative networks including security mechanisms. Such issues are under research.

Cooperative communications consists of schemes and techniques which implement the transmission of data from source station to destination station through one or more intermediate nodes called helpers. These are achieved at the MAC layer level by modifying the MAC protocols as appropriate, and such schemes and the security issues have been discussed [24]. One such MAC protocol is CoopMAC, which describes how the legacy IEEE 802.11 [25] can be modified to implement such cooperative communications, and it is detailed in [26,27].

As the cooperative communication of data is conducted through one or more helpers using CoopMAC, the scheme raises a few potential security issues.

### 3.7. Security Implications in Cooperative Communications in CoopMAC Protocol

As the cooperative communication of data is through one or more helpers using the CoopMAC, the scheme raises a few potential security issues:

- The assister may block services to the sender by not forwarding the data to destination. This requires that the source should ensure every time that the data has been received at the destination through the helper within the given time, by receiving an acknowledgement. The source should have a time-out mechanism, and if the source finds that the data has not been received within the time, the source should send the data through some other helper or send it directly to the destination, knowing that it will be transmitted at a low rate.
- The major security issue is that the intermediate user accesses the sources and the acknowledgement is sent in the name of destination. The sources wrongly assumed the destination person sends that particular acknowledgement. Therefore, CoopMAC is applied to identify the variation between the request, response and acknowledgement (CTS) scheme. The scheme generates the frame used to aware the destination activities in future. If the source does not have any frame value, then the NAV period is applied to detect spoofing activities, and the frame is transmitted to the destination to verify the frame.
- Another problem is a situation where the assistant changes the data and forwards it. The receiver will not be able to identify this and may even send back any sensitive data to source through the helper. This requires an action by the source at the application level that identifies wrong responses and, hence, takes appropriate action.

### 3.8. Security in Presence of CoopMAC

To address the above potential security issues in the cooperative communication using a CoopMAC protocol scheme, the packet header information has to be changed while not violating the IEEE 802.11i standard header format. As a result, the current approach to implementing CoopMAC is incompatible with 802.11i [28]. Integrity checking in both TKIP and AES modes is performed by a message integrity check (MIC) calculated at the source and checked at the destination. This check covers the MAC packet header as well as actual data being transmitted. Thus if the helper changes or introduces new data, the check will fail and so destination will not send acknowledgement (ACK). So the source will identify that there is an issue and so will send the data again. So, in order to solve security concerns in a cooperative network, we must modify the protocol in terms of header data format.

**Figure 8:**
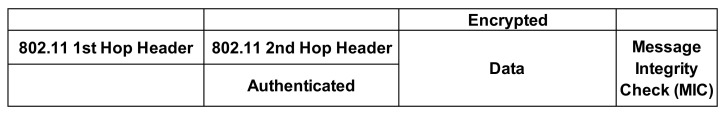
Scheme for two headers.

**Figure 9:**
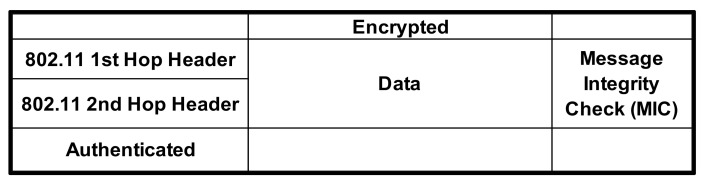
Scheme for single header.

Following a thorough examination of 802.11i and CoopMAC implementation, Salik Makda, Ankur Choudhary, and colleagues proposed two possible solutions in their paper, “Security Implications of Cooperative Communications in Wireless Networks”, in order to create an IEEE 802.11-based CoopMAC architecture [24], as shown in Figure 8 and Figure 9.

## 4. Security Models Using Artificial Intelligence Techniques

As described in previous sections, the challenges in security can be largely addressed in the data link layer, in particular the MAC layer. So, the protocols implemented in the MAC layer should have the intelligence to protect against various types of attacks and implement the defense mechanisms. The intelligence has to implement the defense mechanisms to address the known attacks and to modify or upgrade for newer attacks which we will come across in the future. There can also be a provision for self-learning over time.

Various artificial intelligence models have been used for modeling network security in the past. We can consider implementing such models for WBAN security in individual as well as heterogeneous networks.

Use of Bayesian Networks: In the paper by Peng Xie, Jason H Li, Xinming Ou, Peng Liu, and Renato Levy, “Using Bayesian Networks for Cyber Security Analysis” [29], the authors have presented their work on modeling cyber security considering that cyber security attacks are uncertain and, hence, have to be modeled accordingly. The authors have effectively used previous work in this area in [30,31,32,33,34].

Use of Neural Networks: The paper, “Network Based Intrusion Detection Using Neural Networks” [35], by Alan Bivens, Chandrika Palagiri, Rasheda Smith, Boleslawszymanski, and Mark Embrechts used the neural network approach for modelling network-based intrusion detection. Many methods have been used to detect intrusions. In [36,37,38,39,40,41], the authors effectively used and compared previous work in this area.

Use of Game Theory: In their paper, “Game Theory for Network Security”, *IEEE Communications Surveys Tutorials* [42], Xiannuan Liang and Yang Xiao reviewed game theory procedures to resolve the security problems. The security is occurred due to the defense attacks and security measurement modeling. According to the problems, solutions are given two types such as non-cooperative and cooperative game modeling. In [43,44,45,46], the authors effectively used and compared previous work in this area.

### 4.1. Game Theory for Modeling WBAN HetNet Network Security

The effectiveness of the system is evaluated by investigating the defense-attack interactions which are analyzed using the below scenario. First, the attacks are happened in the computer devices or networks, or nodes and systems. Second, the network or system respond to the attackers. The terms below are utilized to investigate aspects of game theory modelling [42].

- The system, which may be either a host, device, node, process or software entity used to collect the data.
- The person who creates the attacks and affects the system performance and causes loss of the data.
- The system is targeted and the attacked continuously.
- Intrusion detection system (IDS) used to monitor the system activities and identify the intrusion activities. The IDS system has an alarm process which helps to identify the attacks.

From the analysis, the system or applications require the security modelling to reduce intermediate attacks and unwanted user activities. The game theory approach has players who are placed on the security activities; here, two players are involved in activities to identify the attacker and to defend. During the analysis, the intrusion detection system detects the attackers because the security game is more important. The created intrusion system reduces unwanted activities and also ensures error-free data transactions.

### 4.2. Restrictions of Existing Models and Proposal for Use of the Models

The limitations of existing game models are discussed in detail in Xiannuan Liang and Yang Xiao’s paper [42]. In spite of their limitations, game-theoretic approaches have proven to be effective tools for discussing network safety issues. As a result, it is proposed that game theory be used to effectively model security attacks and counterattacks in WBAN in a heterogeneous network. In this paper, however, an advanced model of game theory security equilibrium (GTSSE) is proposed.

## 5. Security Model Development Using Game Theory and Equilibrium Approach (GTSSE)

### 5.1. Overview of GTSEE

The body sensor network uses the set of sensor devices that collects the patient’s health information, which is processed by using the game theory with a Stackelberg security approach. The GTSSE method works according to the player’s involvement that maintains the patient’s authority. Here, position authority makes the decision for players’ activities and the security established according to the game theory model. The organizer places safety resources and handles all type of errors at various potential targets of WBAN [47].

The leadership model is applied to analyze the data in which the leader move first and their firms are moved. During this process, price function P is utilized to investigate the cost structure. Then, the entire output is computed as P(Q1 + Q2) where the Q1 represents the leader and Q2 represents the follower. Suppose the leader firm has the cost structure C1(Q1). After observing the quantity of the leader, the leader considers how the follower will respond. Based on this, the leader selects a quantity that will maximize its profile or benefits, anticipating that the follower will observe the leader’s action and respond accordingly to select a quantity that will maximize its profit or benefits. When the follower picks up this quantity, the market enters a state of equilibrium.

Stackelberg security equilibrium is introduced to handle multiple patients’ health monitoring instantaneously with the least energy consumption in WBAN. In the case of a heterogeneous network, the WBAN node has to consider itself as a leader and all the other nodes of heterogeneous networks as other players following the leader and potential attackers. Then, the introduced security model attains high security and resolves the privacy issues.

### 5.2. Experiment and Results with GTSSE Approach

This work discusses the secure patient health data transmission in the body sensor networks [48]. The main intention of the work is listed as follows.

- The system uses the game theory approach to manage the data security while also monitoring the patient health. The collected information is interconnected with personal system and the stores the information in data server.
- Second, the system uses the Stackelberg security equilibrium approach that helps to analyze the patient health monitoring with mathematical model.
- At last, the Birkhoff–von Neumann theory is applied to reduce the response time and increase the high security while exchanging data using body sensor networks.

The discussed game theory approach compared with the existing methods such as batched group key management (BGKM) [49] and smart wearable sensor-based health monitoring (SWS-HM) [50]. The discussed system is executed using the NS2 simulation tool, and the effectiveness is evaluated in terms of energy consumption and security factors. Thus, the introduced system ensures the minimum response time and reduces the information loss with high data delivery rate.

The security ratio is defined as the ratio of the number of patient data that are securely transferred to the total number of patient data. The security ratio based on the patient’s health information is generally measured in terms of percentage (%) and is formulated mathematically as

$$\mathrm{security}\;=\frac{\begin{array}{c} \mathrm{number}\;\mathrm{of}\;\mathrm{patient}\;\mathrm{data}\;\mathrm{that}\;\mathrm{are}\;\mathrm{securely}\;\\ \mathrm{transferred}\;\mathrm{to}\;\mathrm{the}\;\mathrm{destination}\; \end{array}}{\mathrm{total}\;\mathrm{number}\;\mathrm{of}\;\mathrm{patient}\;\mathrm{data}}$$

The method is said to be more efficient when the security ratio of patient’s health information is higher.

**Figure 10:**
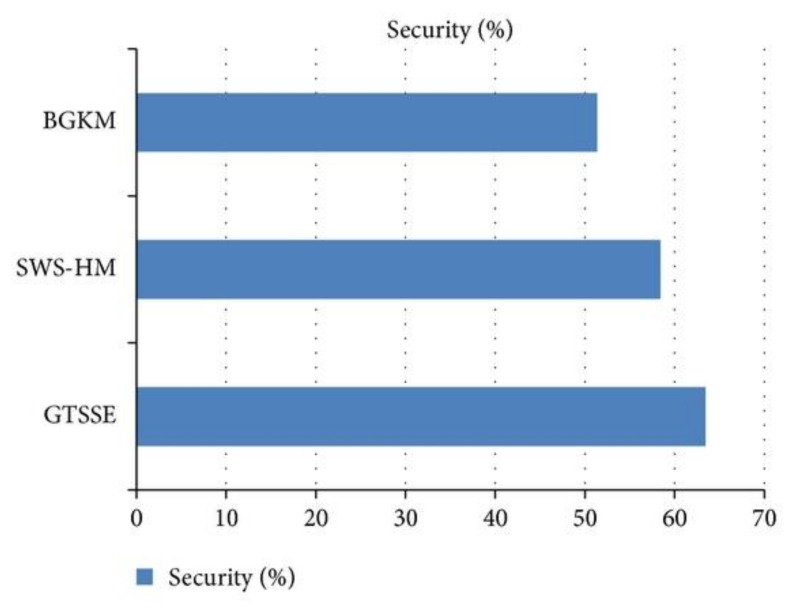
Result of security ratio for GTSSE model compared to other existing models.

Figure 10 shows the security ratio based on patient’s health information with respect to 70 nodes (i.e., patients) during simulation settings at different time intervals. As depicted in the figure, with the increase in the number of nodes, the security rate is also increased. However, when compared to the state-of-the-art works, the security is comparatively better than the two other methods because the proposed GTSSE mechanism uses organizer decision in WBAN and handles the information through game theory approach. So, the security is improved by 7.89% when compared to SWS-HM and 26.95% compared to BGKM, respectively [48].

## 6. Artificial Intelligence Techniques for Wireless Body Area Networks

With advancements in artificial intelligence, AI is emerging as a powerful technique in applying in the area of WBAN. Some of the recent studies and surveys conducted in this area are given here.

### 6.1. AI-Driven Wireless Body Area Networks

In WBAN, the network performance is significantly impacted by human body activities. It was found that the variation of RSSIs (radio signal strength indicator) is dynamically changed when the person is walking, running and jogging. In view of special characteristics of WBAN, AI techniques can be used for various applications such as node localization, sensor fusion, routing and clustering, scheduling, security, QoS and dynamic spectrum access. Honggang Wang et al. have described the various such applications in WBAN [51].

Some of the current challenges posed by emerging Wireless Body Area Networks (WBAN), a key component of smart and connected health (SCH):

○ Network traffic generated is streamed into the network and has different Quality of Service (QoS) requirements. So, the network must become more intelligent, agile, flexible, and programmable using AI techniques. Some of the possible solutions are:
○ Self-configuration: Software-defined networks (SDN), network function vitalization (NFV), and network automation.
○ Self-optimization: Network utilization should be maximized, and all the networks resource should be fully utilized.

In view of the challenges above, some of the AI-assisted approaches for wireless communication are [51]:

○ Network performance improvement: The network performance is significantly impacted by human body activities such as walking, running and jogging, The variation of RSSIs (radio signal strength indicator) is dynamically changed due to these human body activities. So, this information could be very useful for us to configure the networks and optimization. So, this motivates the research on machine learning-based WBANs by collecting the information of the human body and learning to optimize the network performance.
○ Node Localization: In WBAN, location information of the node is important to detect and record the place of events, or to route packets toward the desired location. Genetic algorithm (GA) and swarm intelligence (SI)-based AI techniques are useful for node localization.
○ Sensor Fusion: Sensor fusion is the process of aggregating the data fetched from multiple sensor nodes. This aggregation process may take place at a hub, server, or cloud based on the type of the network structure. Several sensor fusion schemes based on GA, fuzzy logic (FL), re-enforcement learning (RL), and artificial neural networks (ANN) AI techniques have been used for wireless sensor networks (WSN).
○ Routing and Clustering: Routing is the process of determining a connected path between the desired source and destination node for delivering a message. Generally, at the first layer, the sensor nodes form a star topology with the center hub, and thus, a hub-to-node connection is a single hop. However, the routing table requires more memory as the number of sensor node increases. At the second layer, a server can be connected with a number of WBANs, and thus, the server-to-node connection is a two-hop link. At the third layer, multiple server connects to the cloud server. Moreover, a WBAN may move from one server to another, and thus, the involvement of mobility makes the routing more complicated and computationally expensive in terms of computation speed and memory size. A few AI-based algorithms for wireless sensor networks (WSNs) can be used for this. The AI-based routing algorithms are mainly based on EA (evolutionary algorithm), RL, SI, ANN, and fuzzy logic.
○ Scheduling: In order to acquire and transmit data, the sensor node switches between active and sleep mode periodically to save energy. Scheduling algorithms [7] schedule active and sleep time of the sensor nodes in order to trade-off between service quality and sensor battery lifetime.
○ Dynamic Spectrum Access: In the CR-based WBAN, the hub acts as the cognition engine (CE). It performs spectrum sensing and shares the resource with coexisting networks. The CE gathers information regarding the operating environment, capability, and characteristics of the radio. Then, it makes a decision, changes the operation parameters of the radio based on the collected information, and learns the impact of these actions on the performance of the radio. Some ANN-based techniques have developed in spectrum sensing and dynamic spectrum access. Few AI techniques including artificial neural networks (ANNs), metaheuristic algorithms, hidden Markov models (HMMs), rule-based systems, ontology-based systems (OBSs), and case-based systems (CBSs) can be used to in CE implementation.

### 6.2. AI-Driven Adaptable, Reliable and Sustainable Approach

In WBAN, there is need for empowering sensor-based wearable techniques in data collection, data monitoring and healthcare diagnostics. They can be achieved simultaneously by applying AI techniques in the strategies for protocol layers, data routing, battery lifetime, energy optimizations and reliability. Noman Zahid et al. have proposed a framework based on AI techniques to achieve this. They have proposed an Adaptive Transmission Data Rate (ATDR) and Self-Adaptive Routing Algorithm (SARA) to optimize the energy over the dynamic wireless channel [52].

Adaptive Transmission Data Rate (ATDR): Here, we can take advantage of the channel dynamic due to large temporal variations. This algorithm keeps track of the overall energy drain until completion of the task. It works on the average constant energy consumption by varying the active time of the sensor node to optimize the energy over a dynamic wireless channel. For channel estimation and calculation of dynamics, an AI-driven regression method is used to intelligently predict the channel dynamics for the next time slot [52].

Self-Adaptive Routing Algorithm (SARA): Here, in this algorithm, network, transport, MAC and physical layers share the information to optimize the transmission energy and to attain a better QoS with minimum data loss. The SARA algorithm does not use the routing table and updated routing information. The idea is to adopt the dynamic source routing (DSR) strategy with an energy-efficient and shortest possible path. It explores the path from source to destination, by broadcasting the initial packet containing the information about source and destination. For the suitable energy efficient routing path selection, four entities have been considered, namely, residual energy of the relay node, acceptable received signal strength indicator (RSSI), minimum transmission power, and shortest path from source and sink node [52].

### 6.3. Application of Machine Learning Algorithms for IoT

Fadi Al-Turjman et al. has published a survey paper reporting numerous ways machine learning (ML), a sub field of artificial intelligence (AI) is used to benefit the WBAN, the design factors that are considered when implementing the ML algorithms, and the communication technologies used in connecting wearable WBAN in the IoT era. [53]. They are summarized in Table 4.

### 6.4. Trust Management in WBN with AI Techniques

Samiha Ayed et al. has conducted a survey on the various challenges in WBAN in trust management in two categories, namely, intra-WBAN and inter-WBAN. They have proposed a new classification of the existing approaches based on a set of criteria: trust properties, trust objectives, trust techniques and trust computation methods. They have recommended, as a future direction, that to calculate the trust value, more theories in AI such as Dempster–Shafer could be used. AI techniques such as game theory, neural networks and Q-learning can strengthen the trust management framework Additionally, machine learning algorithms, for example, decision tree, SVM, k-nearest neighbor and random forest, will provide the data classification, allowing more accurate prediction of the trust scores and other measures and performing continuous learning and continuous improvement of the predictions [54].

The paper pinpoints best practices that may be useful to build and develop a reliable trust management framework for WBAN. These best practices take into account the limits of existing approaches and give practical recommendations about different steps of the trust management process [54].

**Figure 11:**
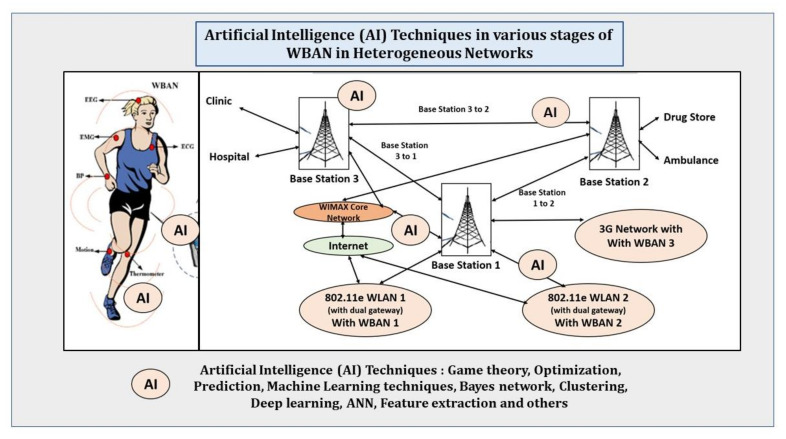
AI techniques in various stages of WBAN in the heterogeneous network.

- Trust models for WBAN have to be independent of medical sensor constraints related to memory usage, computation performance and transmission delay.
- The trust management framework should be an attack-driven model. It has to be directly correlated to the attack detection. Moreover, when a detection is successfully performed, the calculation process has to be updated based on that event. Thus, we evolve towards an intelligent and autonomous trust management framework.
- The trust models should be context aware. They should be dynamic, event driven and continuously updated. The network evolution and the nodes’ behavior changes within time have to be supervised and directly correlated to the reputation and trust calculation processes.
- The trust framework should consider the different trust computation modules to ensure an accurate evaluation of the trust value. During this process, we have to consider the application requirements, the different trust properties, the network topology and behavior. The trust calculation process has to be history dependent including positive and negative past feedbacks.
- The complexity of the calculation process should be low in order to not impact the QoS of the network, especially in the context of medical applications where the exchanged data is very critical and sensitive. The real time characteristic of such applications has to be preserved [54] (Figure 11).

## 7. Conclusions

In summary, the challenges of security in a heterogeneous network can be best addressed by implementing defense mechanisms in the MAC layer of WBAN network communication, addressing current attacks as well as adapting easily to attacks which we will come to know in the future.

Among the various game theory models, the Stackelberg security approach attains the maximum security while transferring data in a sensor network. Various artificial intelligence models have been used for modeling network security in the past. A figure given here summarizes the various stages where AI techniques can be applied. We can consider implementing such models for WBAN security in individual as well as heterogeneous networks.

## Figures and Tables

**Table 1:** “WBAN Security attacks and defences” [3].

Layers	DoS Attacks	Defenses
Physical	Jamming	Lower duty cycle, spread-spectrum, mode change, region mapping and priority messages
	Interfering	Hiding and temper proofing
Link	Smash	Error correction code
	Unfairness	Small frames
	Collapse	Limitation rate
Network	Negligence and greediness	Searching and redundancy
	Homing	Encryption
	Misdirection	Monitoring authorization
	Black holes	Redundancy, observing and authentication
Transport	Flooding and de-synchronization	Dilemmas between clients and authentication

**Table 2:** IEEE 802.14.6 standard-related security.

Name	Explanation	Access Control	Confidentiality	Frame Integrity	Sequential Freshness
Null	No security				
AES-CBC-MAC-32	MAC-32 bit	✓		✓	
AES-CCM-32	MAC-32 bit and Encryption	✓	✓	✓	✓
AES-CTR	CTR and Encryption				
AES-CCM-64	MAC-64bit and Encryption	✓	✓	✓	✓
AES-CBC-MAC-64	MAC-64bit	✓		✓	
AES-CCM-128	MAC128bitand Encryption	✓	✓	✓	✓
AES-CBC-MAC-128	MAC-128 bit	✓		✓	

**Table 3:** Security in IEEE 802.15.6 standard.

Level-0	Insecure communication	Here, data has been broadcasted in an unsafe frame, which means no proper security mechanism is followed to maintain privacy, confidentiality, integrity and authentication.
Level-1	Authentication only	Here, data is transmitted only in a secured manner, but this process not support the privacy and confidentiality.
Level-3	Encryption and authentication	Data is transmitted in secured authentication and encryption frames, addressing all problems not covered in the above levels 0 and 1 (see Figure 3). Every time a nodes enter the network, the security is maintained with the help of master key, new key, group temporal key and pairwise temporal key. These keys help to achieve the multicast communication.

**Table 4:** Application of Machine Learning Algorithms for IoT.

No.	Models	Inputs	Processing	ML Algorithms	Outputs
1	Traffic Profiling	Backbone, Wireless, Mobile networks	Data Capture: (tcpdump, etc.), Data process: (flow extraction, etc.)	Clustering, Bayesian, Frequent item set mining, etc.	Traffic patterns, Traffic engineering, App identification, Security intelligence, etc.
2	Device identification model	PC/Laptop, Mobile phone, sensors, network camera, IoT device	Data capture: Sensors data, network trace, etc., Data Process: feature extraction, etc.	Clustering, kNN, k-means, SVM, etc.	Unique device identification, adverting, network/security engineering, etc.
3	IoT Security Model	Gateway, device, controller	Data capture: traffic, signal, events, configuration, Data Process: flow, feature extraction, etc.	ANN, SVM, Bayes network, Decision tree, k-means	Intrusion detection, anomaly, privacy, authentication,
4	Edge computing in IoT network model	Sensors, edge devices, cloud	Data capture: Sensor data, traffic data, etc. Data process: feature extraction, etc.	Clustering, Bayesian, SVM, Deep learning, Markov, etc.	Intrusion detection, image detection, diseases identification, traffic engineering, etc.
5	Software-defined networking in IoT network model,	Sensors, network devices, controller	Data capture: sensors data, traffic data, etc. Data process: flow, feature extraction, etc.	Clustering, neural network, SVM, Bayesian, etc.	Intrusion detection, traffic management, fault detection, DDoS attack detection, etc.
6	IoT application model	Wearable devices, mobile phone sensors, network camera, wireless sensor network	Data capture: vital signs, environment data, etc. Data process: feature extraction, etc.	Decision tree, logistic regression, SVM, Markov model, Bayes network, clustering, random forest.	Human health condition, human activity, fraud detection, object detection

## Data Availability

Not applicable.
